# Serum TRAIL levels increase shortly after insulin therapy and metabolic stabilization in children with type 1 diabetes mellitus

**DOI:** 10.1007/s00592-015-0731-2

**Published:** 2015-04-12

**Authors:** Gianluca Tornese, Veronica Tisato, Lorenzo Monasta, Liza Vecchi Brumatti, Giorgio Zauli, Paola Secchiero

**Affiliations:** 1 Institute for Maternal and Child Health - IRCCS “Burlo Garofolo”, via dell’Istria, 65/1, 34137 Trieste, Italy; 2 Department of Morphology, Surgery and Experimental Medicine and LTTA Centre, University of Ferrara, Ferrara, Italy

**Keywords:** TRAIL, Type 1 diabetes mellitus, Ketoacidosis, Metabolic status

## Introduction

TNF-related apoptosis-inducing ligand (TRAIL) is a member of the TNF superfamily, which plays an important role in regulating cell death and inflammation. Beyond its anti-tumor activity, increasing evidence in animal studies suggests that TRAIL plays a role in the control of autoimmune diseases, and in particular in type 1 diabetes mellitus (T1DM) [1, 2]. In this context, in a previous study carried out in a retrospective cohort of T1DM pediatric patients, we found significant lower levels of circulating TRAIL in T1DM patients with respect to healthy age-matched controls [3]. However, a limitation of our previous study was as follows: (1) the lack of serial serum samples harvested from the same patients at different time post onset and (2) the lack of information about concurrent metabolic status at time of blood sampling.

On these bases, the aim of the present study was to analyze the evolution of circulating TRAIL levels in a pilot group of pediatric patients admitted at Emergency Department for T1DM, from the time of hospital admission throughout the re-establishment of a normal metabolic balance and up to 18 months of clinical follow-up. Moreover, the serum levels of TRAIL in T1DM patients were analyzed in relation to the metabolic status determined at the same times.

## Materials and methods

### Patients and sample collection

A total of 80 blood samples were obtained from 11 pediatric patients (Table 1) admitted for T1DM onset or secondary diabetic ketoacidosis (DKA) at the Emergency Department of the Institute for Maternal and Child Health “Burlo Garofolo” of Trieste (Italy). For all patients, including patient 7, which was overweight (>85th centile) and had high non-fasting C-peptide levels, the presence of diabetes-associated autoantibodies together with the lack of signs of insulin resistance (i.e. acanthosis nigricans), reasonably excluded T2DM, as well as monogenic diabetes. Blood samples were taken at admission and, in case of DKA, serial blood samples were harvested as per protocol until stabilization. Additional blood samples were taken from each patient before hospital discharge and every 3–6 months during the clinical follow-up. Parents provided informed consent to blood sample drawing for research purposes, in accordance with the Declaration of Helsinki of 1975. The study was approved by the Bioethics Committee of the IRCCS “Burlo Garofolo” (Trieste, Italy; RC 18/13).

**Table 1:** Characteristics of the subjects included in the study

Patient	Sex	Age	Pubertal status	BMI	BMI SDS	Admission	DKA^a^	BG	pH	HCO_3_	HbA1c	C-peptide	Insulin requirement
1	F	14.3	Post-pubertal	26.03	+1.42	Secondary DKA	Severe	753	6.96	6	8–64	N/A	0.65
2	M	13.7	In established puberty	20.20	−0.17	Secondary DKA	Mild	291	7.26	14	9.6–81	N/A	0.60
3	M	8.6	Pre-pubertal	14.38	−0.62	New onset	Moderate	302	7.12	13	13.3–122	0.26	0.70
4	M	12.2	In established puberty	14.76	−2.06	New onset	Moderate	567	7.2	10	11.8–105	0.25	0.85
5	F	7.0	Pre-pubertal	18.94	+0.98	New onset	Moderate	343	7.18	11	12.2–110	0.35	0.94
6	F	5.8	Pre-pubertal	13.91	−1.38	New onset	Mild	570	7.34	11.5	10.7–93	N/A	0.97
7	M	12.6	In established puberty	26.05	+1.48	New onset	None	398	7.45	25	8.6–70	2.56	0.20
8	F	11.6	In established puberty	16.22	−1.25	New onset	None	593	7.56	24	11.3–100	0.67	0.75
9	F	16.8	Post-pubertal	16.10	−2.63	New onset	None	394	7.44	25	8.3–67	0.59	0.52
10	M	9.8	Pre-pubertal	14.85	−1.49	New onset	None	270	7.32	17	12.0–108	0.21	0.75
11	M	9.8	Pre-pubertal	16.74	−0.45	New onset	None	367	7.41	24	9.7–83	0.37	0.31

*BMI* body mass index (kg/m^2^), *BMI* *SDS* body mass index standard deviation score, *BG* blood glucose (mg/dl), *HbA1c* glycated hemoglobin (%—mmol/mol)

HCO_3_ (mEq/l), C-peptide (ng/ml), Insulin requirement (U/kg/day)

^a^DKA was defined: “mild”, if pH was 7.2–7.3 and HCO_3_ 10–15 mEq/l; “moderate”, if pH was 7.1–7.2 and HCO_3_ 5–10 mEq/l; “severe”, if pH was <7.1 and HCO_3_ < 5 mEq/l

### Laboratory analyses and TRAIL ELISA assays

Blood samples were collected into heparinized tubes. All biochemical laboratory tests were performed in Burlo clinical laboratories by using standard commercial kits and following manufacturers’ instructions. Patients demographic characteristics, as well as laboratory/clinical data and treatment, were retrieved from medical records. Serum TRAIL was measured on frozen serum aliquots by using a commercially available ELISA kit (R&D Systems, Minneapolis, MN) following the manufacturer’s instructions, as previously described [3].

### Statistical analysis

Differences between values at two different time points were evaluated with a pairwise sign-rank Wilcoxon’s test. Correlation coefficients were calculated with the Spearman’s rank coefficient rho. A *p* value <0.05 was considered statistically significant, after applying a Bonferroni correction if multiple rank-correlations were calculated simultaneously.

## Results

**Fig. 1:**
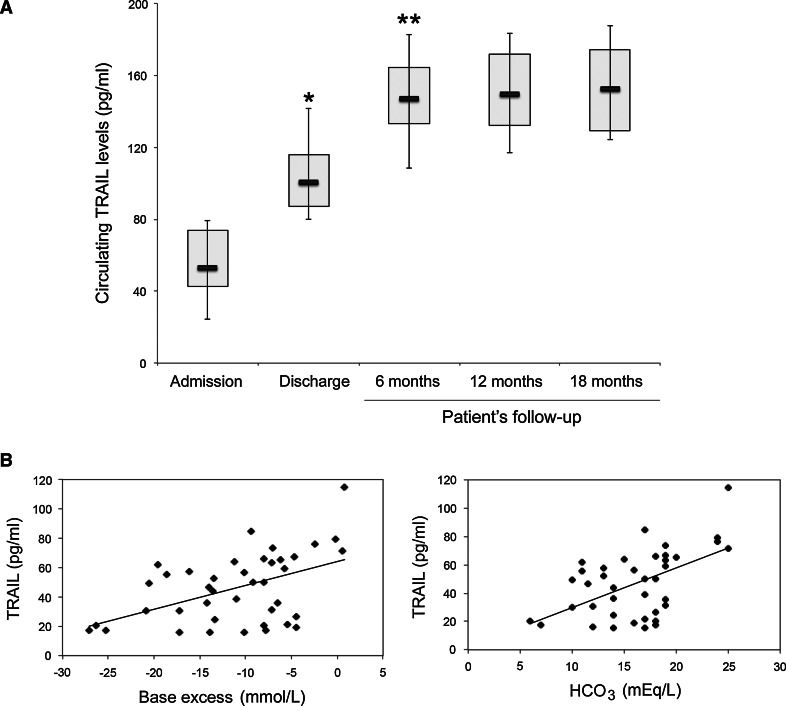
Evolution of the circulating TRAIL levels in relation to the clinical/metabolic status of T1DM patients. **a** Circulating TRAIL levels were monitored in serum samples collected at the indicated time points of the clinical history of the T1DM pediatric patients enrolled in the study (*n* = 11). *Horizontal bars* are median, *upper* and *lower* *edges* of *box* are 75th and 25th percentiles, *lines* extending from *box* are 10th and 90th percentiles. *p < 0.01 compared to admission; **p < 0.05 compared to discharge. **b** Correlation analyses between TRAIL circulating levels and clinical parameters related to the metabolic status of patients. Positive correlation between TRAIL and base excess (BE) as well as between TRAIL and bicarbonate (HCO_3_) are shown. Correlation coefficients, calculated by Spearman’s analysis, are reported for each correlation in the text

The main demographical and clinical characteristics of the pediatric patients enrolled in the present study are summarized in Table 1. Comparative analysis of the circulating TRAIL levels, showed median TRAIL level at admission of 52.8 pg/ml (mean ± SD 57.2 ± 27.1 pg/ml), with the lowest levels measured in the patients with DKA. Of interest, in all patients included in the study, we documented a significant increase of TRAIL levels (*p* < 0.01, using the sign-rank pairwise Wilcoxon test) at the time of discharge, with a median value of 100.3 pg/ml (mean ± SD 109.8 ± 33.9 pg/ml) (Fig. 1a). Of interest, a further significant (*p* < 0.05) increase was documented after 6 months, reaching median values of TRAIL of 147.3 pg/ml (mean ± SD 146.1 ± 37.1 pg/ml) that were maintained without significant modulations also in the subsequent time points/assessments up to 18 months (Fig. 1a).

As for clinical protocol, in the patients with DKA (*n* = 6) upon admission between 5 and 10, blood samples were taken for each subject before reaching normalization of venous blood gas (VBG) and/or blood glucose (BG). This allowed us to monitor TRAIL levels also at the time points corresponding to metabolic stabilization (median time between admission and stabilization of 15.5 h; IQR 10.1–20.9). Analysis of TRAIL levels in relationship with all the clinical/metabolic parameters reported in Table 1 revealed a significant (*p* < 0.05) correlation only between TRAIL levels and: HCO_3_ (Spearman’s rank correlation, *ρ* = 0.4944, *p* = 0.0014; Fig. 1b), BE (*ρ* = 0.4405, *p* = 0.0044; Fig. 1b), pH (*ρ* = 0.368, *p* = 0.0193), pCO_2_ (*ρ* = 0.3962 *p* = 0.0114).

## Discussion

In both newly diagnosed T1DM and in secondary DKA pediatric patients, we documented a significant increase of serum TRAIL levels after short-standing insulin treatment has been established. Moreover, the circulating levels of TRAIL correlated with the metabolic parameters. To our knowledge, this is the first report demonstrating a rapid modulation of circulating TRAIL during the first hours after T1DM onset and/or DKA and its relationship with the metabolic status, further supporting a link between TRAIL and T1DM. In particular, even if carried out in a small cohort, our pilot study can strongly suggest the link between TRAIL and the metabolic status, because (1) a worst metabolic status (documented by HCO_3_, BE, pH and CO_2_ assessments) is associated with lower TRAIL levels, and (2) improvement in TRAIL levels is seen shortly upon metabolic stabilization. A potential molecular mechanism that links metabolic state and circulating levels of TRAIL might be represented by C reactive protein (CRP). Indeed, it has been shown that CRP is elevated in children with newly diagnosed T1DM and DKA crisis and that it likely enhances inflammation process by modulating a variety of cytokines [4]. In particular, it has been reported that CRP is able to up-regulate pro-inflammatory cytokines IL-6, IL-8 and TNF-α, and also to decrease IL-10 secretion. Likewise, we have previously documented that TRAIL expression and release is down-regulated by CRP [5]. Although the mechanism underlining the down-regulation of TRAIL in T1DM should be further investigated, these findings suggest how the metabolic endangerment, rather than autoimmunity, is clearly linked to the decrease in the circulating levels of TRAIL.

## References

[1] Di Bartolo BA, Chan J, Bennett MR, Cartland S, Bao S, Tuch BE, Kavurma MM (2011). TNF-related apoptosis-inducing ligand (TRAIL) protects against diabetes and atherosclerosis in Apoe−/− mice. Diabetologia.

[2] Zauli G, Toffoli B, di Iasio MG, Celeghini C, Fabris B, Secchiero P (2010). Treatment with recombinant tumor necrosis factor-related apoptosis-inducing ligand alleviates the severity of streptozotocin-induced diabetes. Diabetes.

[3] Tornese G, Iafusco D, Monasta L, Agnoletto C, Tisato V, Ventura A, Zauli G, Secchiero P (2014). The levels of circulating TRAIL at the onset of type 1 diabetes are markedly decreased in patients with ketoacidosis and with the highest insulin requirement. Acta Diabetol.

[4] Karavanaki K, Kakleas K, Georga S, Bartzeliotou A, Mavropoulos G, Tsouvalas M, Vogiatzi A, Papassotiriou I, Karayianni C (2012). Plasma high sensitivity C-reactive protein and its relationship with cytokine levels in children with newly diagnosed type 1 diabetes and ketoacidosis. Clin Biochem.

[5] Secchiero P, Rimondi E, di Iasio MG, Agnoletto C, Melloni E, Volpi I, Zauli G (2013). C-reactive protein downregulates TRAIL expression in human peripheral monocytes via an Egr-1-dependent pathway. Clin Cancer Res.

